# Synthesis, Fabrication, and Characterization of Functionalized Polydiacetylene Containing Cellulose Nanofibrous Composites for Colorimetric Sensing of Organophosphate Compounds

**DOI:** 10.3390/nano11081869

**Published:** 2021-07-21

**Authors:** A K M Mashud Alam, Donovan Jenks, George A. Kraus, Chunhui Xiang

**Affiliations:** 1 Department of Apparel, Events, and Hospitality Management, Iowa State University, Ames, IA 50011, USA; mashud@iastate.edu or; 2 Department of Chemistry, Iowa State University, Ames, IA 50011, USA; djenks@iastate.edu (D.J.); gakraus@iastate.edu (G.A.K.)

**Keywords:** organophosphate (OP), polydiacetylene (DA), nanofibrous (NF) composite, electrospinning, colorimetric sensor, regenerated cellulose (RC), chemical protective clothing (CPC)

## Abstract

Organophosphate (OP) compounds, a family of highly hazardous chemical compounds included in nerve agents and pesticides, have been linked to more than 250,000 annual deaths connected to various chronic diseases. However, a solid-state sensing system that is able to be integrated into a clothing system is rare in the literature. This study aims to develop a nanofiber-based solid-state polymeric material as a soft sensor to detect OP compounds present in the environment. Esters of polydiacetylene were synthesized and incorporated into a cellulose acetate nanocomposite fibrous assembly developed with an electrospinning technique, which was then hydrolyzed to generate more hydroxyl groups for OP binding. Scanning electron microscopy (SEM), Fourier-transform infrared spectroscopy (FT-IR), Instron^®^ tensile tester, contact angle analyzer, and UV–Vis spectroscopy were employed for characterizations. Upon hydrolysis, polydiacetylene esters in the cellulosic fiber matrix were found unaffected by hydrolysis treatment, which made the composites suitable for OP sensing. Furthermore, the nanofibrous (NF) composites exhibited tensile properties suitable to be used as a textile material. Finally, the NF composites exhibited colorimetric sensing of OP, which is visible to the naked eye. This research is a landmark study toward the development of OP sensing in a protective clothing system.

## 1. Introduction

Organophosphate (OP) toxins are a family of highly hazardous chemical compounds included in nerve agents and pesticides [1,2]. OP pesticides alone have been reported in over 3 million cases of OP poisoning every year, causing more than 250,000 annual deaths [2]. OPs are dangerously toxic and prevalent in the environment; thus, there is a critical need for the development of technologies for the detection and degradation of these colorless and odorless threats to human beings, animals, aquatic species, and the environment [1,3,4].

Conventional analytical techniques, namely gas chromatography (GC), high-performance liquid chromatography (HPLC), and mass spectrometry (MS), provide adequate selectivity and sensitivity and are considered to be the most reliable detection techniques [5,6]. However, these techniques require expensive instruments, detailed experimental conditions, multi-step sample preparation, and well-trained operators [6]. Among other techniques, simple and low-cost enzyme inhibition is limited by low specificity; enzyme-linked immunosorbent assay (ELISA) requires costly and time-consuming antibody preparation and is limited by the specificity and poor stability of the enzymes or antibodies. Moreover, none of these techniques are suitable for equipment-free, on-site, or in situ detection with sensitivity and specificity [6,7]. Therefore, significant challenges still remain in exploiting rapid, reliable, specific, and sensitive detection of OP toxins, particularly with equipment-free real-time sensor systems [6].

Optical sensors, as an alternative detection method, provide a facile, rapid, and low-cost approach for sensitive detection of OPs based on colorimetric, fluorescence, UV–Vis, Raman, or other chemiluminescence signal variations that employ functional materials as a sensing probe, whose absorption or emission changes due to the presence of OP [1,8]. Among them, colorimetric sensors have been attractive due to their ease of use, quick response, equipment-free detection, and cost-effectiveness, because a chemical interaction causes a color change that can be visualized by the unaided eye [8,9]. The direct visualization of the response signal eliminates the requirement of any additional transduction element, making these sensors highly interesting for the development of smart devices for point-of-care applications [10]. However, the key challenge for fabricating a colorimetric sensing platform is transforming the response behavior into a visual color change. Conjugated polymers (CPs) have gained great interest in the development of colorimetric sensors due to their fascinating optical, optoelectronic, and magnetic properties [11].

Among the CPs, polydiacetylenes (PDAs), a class of amphiphilic polymers composed of a carboxylic head and an alkyl tail, are especially attractive due to their ability to exhibit a blue-to-red color transition visible to the naked eye when they are subjected to external stimuli [2,12]. PDAs are formed via 1,4 addition photopolymerization of self-assembled diacetylene (DA) monomers, such as 10,12 pentacosadiynoic acid (PCDA), resulting in a highly ordered conjugated backbone [2,13]. PDAs exhibit a blue color after photopolymerization with maximum absorption (λ_max_) at 640 nm and show a color shift to red having maximum absorption (λ_max_) at around 540 nm upon exposure to an external stimulus [9,13]. Researchers nowadays share a common opinion that the shortening of the conjugation length and the widening of the HOMO–LUMO energy gap due to a change in the configuration of the PDA backbone in the presence of external stimuli brings out the color change in PDA [2,5]. PDAs have been functionalized via the modification of the terminal carboxylic acid with suitable functional groups for numerous sensing applications, such as temperature and pH [14], cations [15], anions [16], surfactants [17], gas molecules [18], glucose [19], enzymes [20], amino acids [21], viruses [22], bacteria [23], and OP [2,5]. In 2012, Lee et al. studied functionalized PDA with oxime moiety for the detection of OP in a basic (pH ˃ 9.0) liquid medium [5]. Zhang et al. functionalized PDA with pralidoxime for the detection of the OP agent malathion in liquid media [2]. However, any attempt to fabricate a solid-state bulk, micro, or nano structure is absent in the literature. Therefore, this study is dedicated to the development of a solid-state nanofibrous (NF) composite incorporated with PDA ester having aldehyde functionality. The NF composite-based sensing platform could be promising due to the nanoscale size, light weight, porous structure, high surface area-to-volume ratio, and high target specificity in monitoring OPs with greater precision and lower detection limits [24,25].

Electrospinning has been a versatile technique for fabricating nanoscale filament fibers with high specific surface area and high porosity using high electrostatic force [26]. Jeon et al. exhibited superior sensitivity of PDA electrospun nanofiber structures over thin films prepared from the same solution [18]. However, due to the relatively low viscosity of PDAs in solution, electrospinning without any high viscosity supporting polymer (matrix polymer) is highly challenging [13,27,28]. For this study, regenerated cellulose (RC) was selected as the matrix polymer due to the presence of a large number of exposed hydroxyl (OH^−^) groups on the composite structure, which favors the reaction with OP to enhance the sensitivity of the composites. Due to the difficulty of the direct electrospinning of cellulose, cellulose acetate (CA) was chosen as the prepolymer for electrospinning, which was later hydrolyzed to RC via chemical treatment. RC is high in adsorption capacity, and it is extremely suitable for applications such as adsorption in purification and filtration [29,30], food packaging [31], and biomedical applications [32,33]. Moreover, RC retains chemical resistance to almost all organic solvents and aqueous solutions in a broad pH range, making it a suitable choice for sensing materials.

This article presents the synthesis of aldehyde-functionalized PCDA ester following a green approach and fabrication of the novel electrospun cellulosic nanofibrous composites containing aldehyde-functionalized PCDA ester (PCDA-HBA) for the colorimetric detection of OP toxins. The effect of the regeneration of cellulose (RC) via hydrolysis treatment on the chemical structure of PCDA-HBA was studied, particularly if PCDA-HBA could retain its structural integrity after chemical hydrolysis. The NF composites were characterized with SEM, FT-IR, a contact angle analyzer, and Instron^®^ tensile tester. Finally, the RC-PDA-HBA nanocomposite fiber sensors were tested against OP for sensing.

## 2. Materials and Methods

### 2.1. Materials

10,12-Pentacosadiynoic acid (PCDA, 98%) was purchased from GFS Organics (Columbus, OH, USA). Cellulose acetate (CA) with 39% acetyl content and a number-average molecular weight ($\bar{M_{n}}$) of 30 kDa, determined by gel permeation chromatography (GPC), was purchased from Sigma-Aldrich (St. Louis, MO, USA). 2,4-dihydroxybenzaldehyde (98%), 4-(Dimethylamino) pyridine (DMAP, ≥99%), *N,N*′-dicyclohexylcarbodiimide (DCC, 99%), and sodium hydroxide (≥97%) were also purchased from Sigma-Aldrich. Certified ACS grade acetone (≥9.9%), methylene chloride (99.8%), hexane (95%), tetrahydrofuran (THF, 99.9%), and ethyl acetate (99.5%) were purchased from Fisher Chemical (Fair Lawn, NJ, USA). Ultrapure deionized water was purchased from VWR life sciences (Radnor, PA, USA).

### 2.2. Methods

#### 2.2.1. Synthesis of Aldehyde-Functionalized PCDA Ester (PCDA-HBA)

A total of 1.53 gm (4.08 mmol) of 10,12-Pentacosadiynoic acid (PCDA), 0.125 gm (1.02 mmol) of dimethylaminopyridine (DMAP), and 0.565 gm (4.09 mmol) of 2,4-dihydroxybenzaldehyde were combined in a light-protected round-bottom flask. The mixture was dissolved in 100 mL of dichloromethane (DCM) and sparged with argon. A 15 mL solution containing 0.43 g (2.08 mmol) of N, N′-dicyclohexylcarbodiimide (DCC) was slowly added to the solution by syringe and stirring continued on a hot plate stirrer for 24 h at room temperature. Dicyclohexylurea, the reaction byproduct, was filtered off by gravity filtration. The solvent was removed under vacuo on a rotary evaporator (BUCHI Rotavapor, Model R-200, BUCHI Co., New Castle, DE, USA) and yielded a tan solid. The residue was purified by column chromatography with 10:1 hexane: ethyl acetate, and the desired diacetylene monomer PCDA-HBA was obtained as a white solid. The use of DCC-based coupling led to a much cleaner product compared to the oxalyl chloride-based procedure followed by other researchers [5]. The structure was confirmed with a nuclear magnetic resonance (^H^NMR) (Varian MR 400, Varian, Palo Alto, CA, USA) at room temperature and stored in a freezer protected from light and air.

#### 2.2.2. Fabrication of PCDA-HBA Containing Cellulose Acetate Nanocomposite Fibers

##### Preparation of Electrospinning Solution

The electrospinning solution was prepared at various solution concentrations (*w*/*v*) by adding PCDA-HBA with cellulose acetate in a 4:1 molar ratio in acetone according to the design in Table 1. The required amount of PCDA-HBA was dissolved in acetone first, followed by the incorporation of CA into the homogeneous solution of PCDA-HBA. The resulting mixture was kept under constant stirring overnight at room temperature with a Burrell wrist-action^®^ shaker, model-75 (Burrell Scientific LLC, Pittsburgh, PA, USA), to obtain a homogeneous CA-PCDA-HBA solution ready for electrospinning.

##### Electrospinning of CA-PCDA-HBA Nanocomposite Fibers

A 10 mL plastic syringe was loaded with a freshly prepared CA-PCDA-HBA solution and attached to a stainless steel needle with a 0.8 mm inner diameter. A syringe pump (Harvard Apparatus, Holliston, MA, USA) was employed to feed the spinning fluid to the spinning zone at a fixed rate of 1 mL h^−1^ or 3 mL h^−1^, following Table 1. A fixed electric potential of 15 kV was applied, by a DC power supply instrument (Gamma High Voltage Research, Ormond Beach, FL, USA), between the needle tip and the Al foil collector. The needle tip and the Al foil collector were separated by a 10 cm distance. The electrospinning process was conducted for 2 h under ambient conditions to obtain a thick colorless fiber membrane.

##### Deacetylation of CA-PCDA-HBA into RC-PCDA-HBA Nanocomposite Fibers

The deacetylation/hydrolysis of the CA-PCDA-HBA composite membranes was carried out to remove acetyl groups from the composites following a previously reported method [34,35]. In short, a 0.1 M NaOH solution was prepared in deionized (DI) water, which was then used to prepare a deacetylation solution comprising 4:1 NaOH: EtOH. The CA-PCDA-HBA composites sandwiched between two glass fiber meshes were soaked in the deacetylation solution in a glass bowl. A magnetic stirring bar placed on top of the mesh was kept rotating at 150 rpm for 30 h at room temperature on a hot plate. The membranes were then thoroughly rinsed with deionized water to complete neutralization, which was confirmed by pH paper. They were then air-dried overnight, followed by vacuum drying at 50 °C for 24 h to obtain RC-PCDA-HBA composite membranes. The RC-PCDA-HBA composites were then stored in a dark place away from light.

##### Photopolymerization of RC-PCDA-HBA Nanocomposite Fibers

The RC-PCDA-HBA nanocomposite fibers were photopolymerized with 254 nm UV light (Spectroline, Longlife^TM^ filter, New York, NY, USA) irradiation for 3 min on both sides of the fibers [13]. During the UV irradiation, the colorless fibers started turning blue within 30 s, which turned into deep blue RC-PDA-HBA nanocomposite fibers in 3 min.

#### 2.2.3. Characterization of Nanocomposite Fibers

##### Morphology

A field emission scanning electron microscope (FEI Quanta 250 FE-SEM) was employed to study the size and surface morphology of the nanocomposite fibrous membranes. To evaporate any residual solvent or moisture, the membranes were kept under vacuum overnight before SEM imaging. They were then sputter-coated with a 5 nm layer of iridium to improve the conductivity of the samples for improved imaging. Image J software (National Institute of Health, Bethesda, MD, USA) was used to calculate the diameter and distribution of the fibers. The average and distribution of diameters were determined by measuring 50 representative fibers from the SEM images.

##### Confirmation of the Regeneration of RC-PCDA-HBA Nanocomposites

The regeneration of RC-PCDA-HBA from CA-PCDA-HBA was studied through functional group analysis with an Agilent Cary 630 Fourier-transform infrared (FT-IR) spectrometer (Agilent Technologies, Inc., Danbury, CT, USA) equipped with a smart DATR accessory. All the nanocomposites were dried overnight in an Isotemp^®^ (Model 282 A) programmable vacuum oven (Fisher Scientific, Waltham, MA, USA) at room temperature before FT-IR analysis. Before taking the IR of the samples, the air was used as a background to run the experiments. Each sample was scanned 32 times at a resolution of 4 cm^−1^ and an interval of 1 cm^−1^ over the wavenumbers ranging from 750 to 4000 cm^−1^.

##### Contact Angle

A video-based drop shape analyzer (OCA 25, Data Physics, Filderstadt, Germany) was used to measure the static water contact angles of the composites at room temperature following the ASTM standard, ASTM-D7334-08 sessile drop method. A droplet of 2 μL of deionized water was dropped to the composite’s surface from a micro-syringe. The built-in software SCA20 (V.4.5.20) was used to measure the contact angles. The reported values are the average of three readings for each type of composite sample.

##### Tensile Properties

The tensile properties of the RC-PCDA-HBA membranes were tested according to ASTM D638-10 using an Instron 5966 (Instron, Boston, MA, USA) tensile testing machine mounting a load cell of 250 N. Test specimens with dimensions of 75 mm × 10 mm, after conditioning at ambient conditions for a day, were used for a gauze length of 30 mm for the test. The samples were stretched at a crosshead speed of 10 mm min^−1^. Five replications were tested for each sample. The typical stress–strain curves were plotted from the measured load and extension vales. A digital caliper (Electron Microscopy Sciences, Hatfield, PA, USA) was used to measure the thickness of the specimens.

##### UV–Vis Spectroscopy

The colorimetric sensing properties were measured with a UV–Vis spectrophotometer (Nicolet Evolution 300 UV−Vis spectrophotometer, Thermo Scientific, Waltham, MA, USA) at room temperature. To obtain dry, solid NF composite materials, the solvent was removed by drying the composites overnight at 40 °C using a vacuum oven. The absorption spectra of the NF composites were collected in the range of 400–800 cm^−1^ wavelength before and after the treatment with DFP. Representative spectra from three tests were reported.

## 3. Results and Discussion

### 3.1. Synthesis of PCDA-HBA

**Figure 1:**
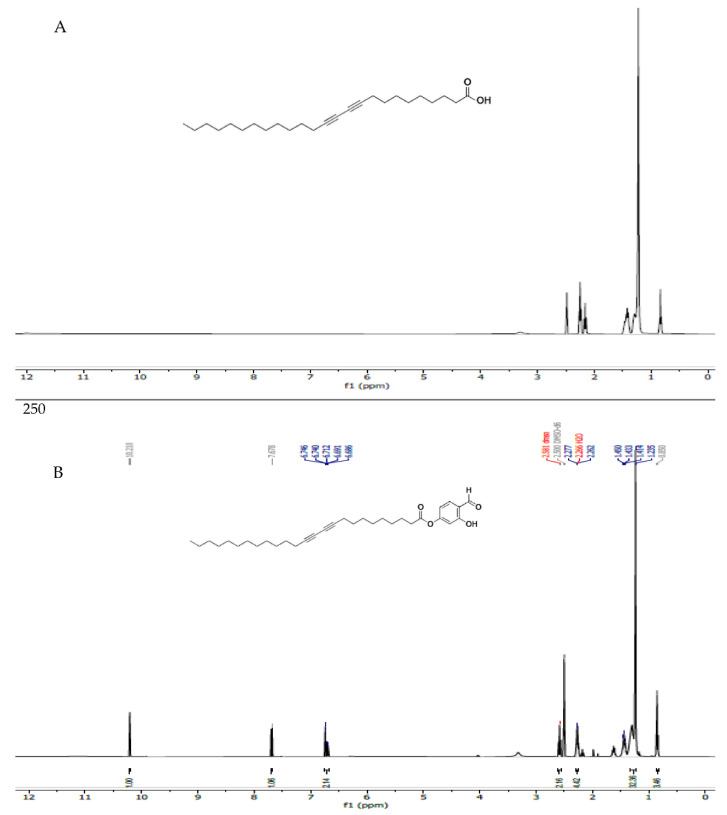
The ^H^NMR spectra of 10,12 Pentacosa diynoic acid (PCDA) (**A**) and PCDA-HBA (**B**).

The ^H^NMR spectra of 10,12 Pentacosa diynoic acid (PCDA) and PCDA-HBA were recorded on a 400 MHz instrument using DMSO-d6 as the solvent (Figure 1). The peaks can be assigned as follows: ^1^H NMR (400 MHz, DMSO-d6): δ 0.85 (t, 3H), 1.23–1.63 (m, 32H), 2.26 (t, 4H), 2.51 (t, 2H), 6.68–6.75 (m, 2H), 7.68 (s, 1H), and 10.21 (s, 1H). The peak at 0.85 ppm belongs to the methyl (-CH_3_) group; the multiple peaks between 1.23 and 1.63 ppm relate to the methylene group (-CH_2_-) of the hydrocarbon chain; the peaks at 2.26 and 2.51 ppm relate to the methylene group adjacent to the alkyne (-C≡) group; the peaks between 6–8 ppm in spectrum B belong to the protons attached to the aromatic ring, and the peak at 10.21 ppm belongs to the aldehyde group. Comparing and calculating spectra A and B, spectrum B confirms the presence of aldehyde-functionalized PCDA ester, PCDA-HBA. The spectra of PCDA and PCDA-HBA are consistent with those in the previously published literature [5].

### 3.2. Fabrication of CA-PCDA-HBA Composite NF

The fabrication of NF composites containing cellulose acetate (CA) via the electrospinning technique is critical and requires precise control over processing parameters. The fabrication and morphology of the fibers have been found to be influenced by factors such as the polymer molecular weight, solution concentration, solution viscosity, conductivity, solvent surface tension, vapor pressure, solubility etc. [13]. For this study, we chose acetone as the solvent because of its low surface tension (22.7 mN/m) as well as its compatible solubility parameter (9.8 J/cm^3^) with CA [36]. The addition of PCDA-HBA with the CA solution in acetone resulted in viscosity reduction of the solution as compared to the pristine CA solution, as has been seen for pure PCDA macromolecules by other researchers [37]. Among the critical electrospinning parameters, applied voltage and tip-to-collector distance, along with ambient conditions, were kept constant during the trial runs. On the other hand, due to their significant effect on fiber size and morphology, polymer concentration and injection speed were investigated to find the optimum electrospinning parameters for the development of CA-PCDA-HBA NF composites.

In order to set up the critical parameters of electrospinning to obtain defect-free regular fibers, we conducted several preliminary experiments designed with varied applied voltage ranging from 10 to 25 kV, and tip-to-collector distance ranging from 5 to 20 cm at different concentrations. Based on these initial experiments, we fixed the electrostatic force at 15 kV and tip-to-collector distance at 10 cm with two levels of injection speed (1 and 3 mL·h^−1^) under ambient conditions, as this combination of parameters was found to be optimal in developing electrospun fiber composites.

It was found from our initial trials that a CA-PCDA-HBA concentration of less than 10% (*w*/*v*) resulted in electrospraying only; no fibers were obtained due to insufficient polymer chain entanglements. Fibers started to form at 12% concentration and above. At 15% concentration, continuous fibers without beads were obtained. Concentrations above 17% were also found not feasible for fiber formation. The high solution viscosity, along with high evaporation of acetone, made the fiber formation difficult due to needle clogging. Therefore, the CA-PCDA-HBA NF composites electrospun over 15% (*w*/*v*) concentration were found the most promising for developing bead-free continuous fibers at nanometer scale.

### 3.3. Characterizations

#### 3.3.1. Morphology of the Nanocomposite Fibers

**Figure 2:**
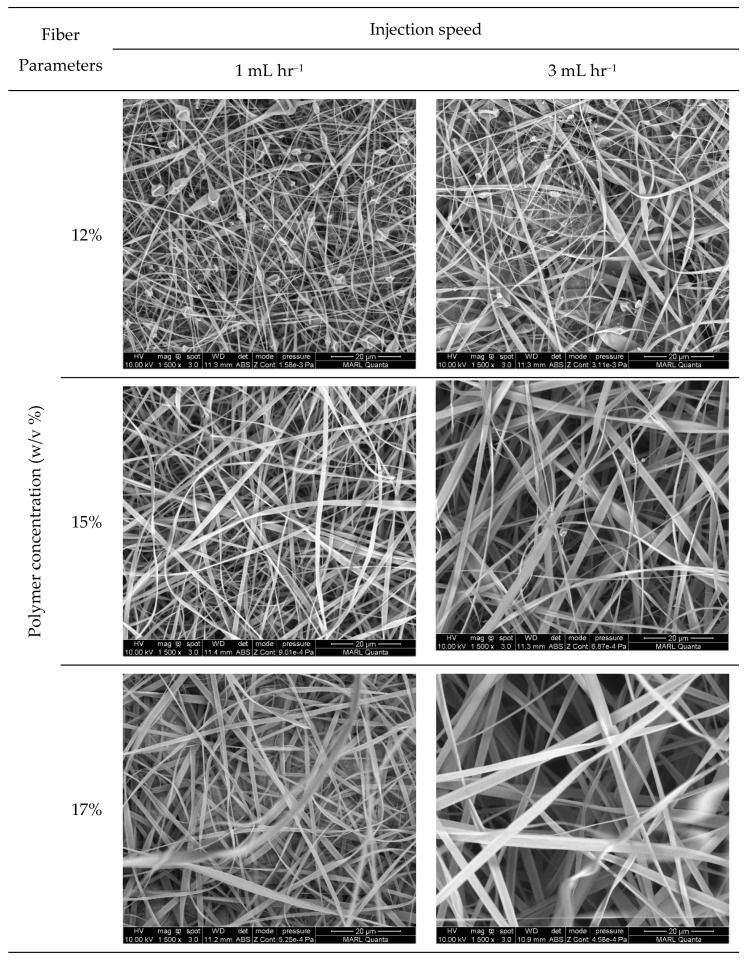
SEM images of electrospun CA-PCDA-HBA composite nanofibers at different polymer concentrations and injection speeds.

**Figure 3:**
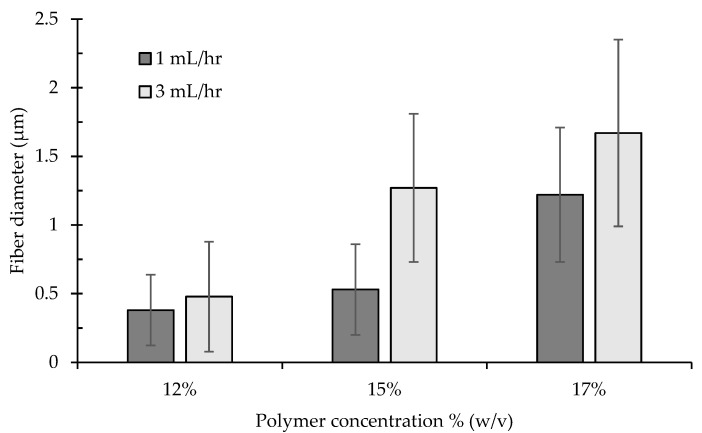
Fiber diameter at various polymer concentrations and injection speeds of the CA-PCDA-HBA nanofiber composites.

Figure 2 exhibits the SEM images of the electrospun nanocomposite fibers developed at three levels of concentration and two levels of injection speed. Figure 3 shows the average diameter of the fibers with the corresponding standard deviation as a function of polymer concentration in the electrospinning fluid. The fibers in the membrane, irrespective of the solution concentration and injection speed, are fluffy and show less inter-fiber adhesion due to the presence of a charged acetate group on the fiber surface [35].

Polymer concentration and injection speed showed profound effect on the size and morphology of the nanocomposite fibers. At 12% (*w*/*v*) concentration, fibers were formed along with spindle-shaped beads and defects at both 1 and 3 mL·h^−1^ injection speed due to the low viscosity of the polymer solution (Figure 2). The formation of beads during electrospinning usually occurs due to insufficient polymer chain entanglement of the solvent molecules. Beads on fiber strings are highly undesirable for sensing applications; therefore, a 12% solution concentration was not suitable for continuous nanofiber development in this study. The increase in polymer concentration helped to cease bead formation. At higher solution concentrations (15 and 17%), sufficient polymer chain entanglement helped the formation of defect-free regular continuous fibers. However, at high polymer concentrations, polymer chain mobility decreases due to high entanglements, leading to increased difficulty in jet extension, favoring thicker fibers. The average fiber diameter of the CA-PCDA-HBA composites at 1 mL h^−1^ was calculated as 0.38 ± 0.26, 0.53 ± 0.33, and 1.22 ± 0.49 µm, respectively, for 12, 15, and 17% concentrations. On the other hand, the average fiber diameter at 3 mL h^−1^ was calculated as 0.48 ± 0.4, 1.27 ± 0.54, and 1.67 ± 0.68 µm, respectively. Other researchers also reported a similar trend of fiber diameter increase in response to the change in polymer concentration [38].

The injection speed has significant effect on the size of the fibers [12]. A low injection speed along with a low concentration of polymer solution promoted beaded fibers (Figure 2). The size of the beads increased at a higher injection speed. Higher injection speed promoted larger fiber diameters due to the infusion of a higher amount of polymer solution to the spinning zone (Figure 3). Therefore, a suitable injection speed along with an optimal polymer concentration is critical for the development of defect-free continuous fiber composites.

#### 3.3.2. Regeneration of CA-PCDA-HBA Nanocomposite Fibers into RC-PCDA-HBA

**Figure 4:**
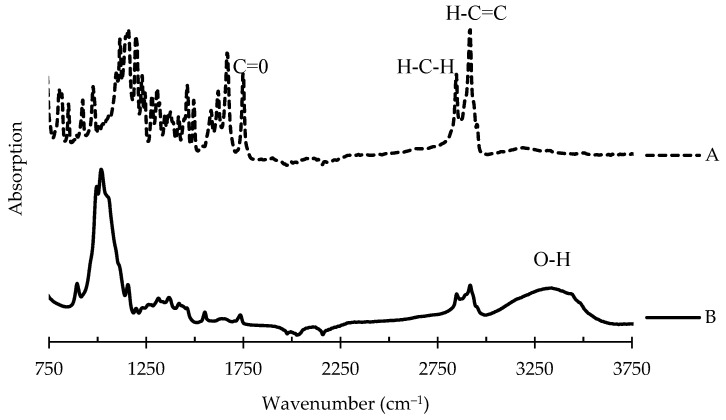
FT-IR spectra of PCDA-HBA crystal (**A**) and regenerated cellulose composite nanofiber RC-PCDA-HBA (**B**).

In this study, PCDA-HBA was mixed with CA to facilitate the fabrication of CA-PCDA-HBA NF composites, and, after electrospinning, the composites were chemically treated to regenerate into RC-PCDA-HBA NF composites. This leads to the question of whether the matrix polymer CA mixed with PCDA-HBA has any effect on conjugated macromolecules, hindering the sensing properties of the NF composites. Therefore, attenuated total reflectance−Fourier-transform infrared spectroscopy (ATR− FT-IR) was used for chemical analysis. Spectrum A of Figure 4 corresponds to the pristine PCDA-HBA crystal, where the following resonance features were interpreted for characterization: IR ν_max_ (cm^−1^): 2914 (aldehydic H-C=C stretch), 2843 (aldehydic H-C-H stretch), 1747 (ester, C=O stretch), and 1460 (aldehydic C−H stretch bending), 1155 (C−O stretch). The resulting resonance features were similar to previously reported results in the PDA literature, and the spectrum was consistent with the anticipated structure of PCDA [9,13]. The two peaks between 2800 and 2950 for the C-H bond, along with the carbonyl (C=O) peak around 1750, indicated the presence of the aldehyde group added to the PCDA structure. The characteristic vibration band in the double bond region, around 1600 cm^−1^ adjacent to the carbonyl bond, indicated the stretching motion of a benzene ring in the structure. The C(sp2)-H bond vibration observed near 3000 cm^−1^ is also indicative of the presence of a benzene ring in the structure.

Spectrum B represents the regenerated cellulose RC-PCDA-HBA NF composites. The following resonance features were observed: ν_max_ (cm^−1^): 3350 (O-H), 2921 (aldehydic H-C=C stretch), 2854 (aldehydic H-C−H stretch), 1740 (ester, C=O stretch), 1430 (aldehydic C−H stretch bending), and 1020 (C−O stretch). The similarities in the IR spectra with the PCDA-HBA crystal confirm that the PCDA-HBA retains its functional groups in the matrix polymer structures even after the deacetylation process (RC-PCDA-HBA). The carbonyl (C=O) peak shift toward a lower wavenumber indicates the decrease in the CO group after hydrolysis. The appearance of a broad absorption peak at 3350 cm^−1^ after deacetylation indicates the development of a large number of hydroxyl (O-H) groups due to the successful deacetylation process, indicating the successful conversion of the CA-PCDA-HBA structure into the RC-PCDA-HBA structure without any damage to the chemical structure of PCDA-HBA [35].

**Figure 5:**
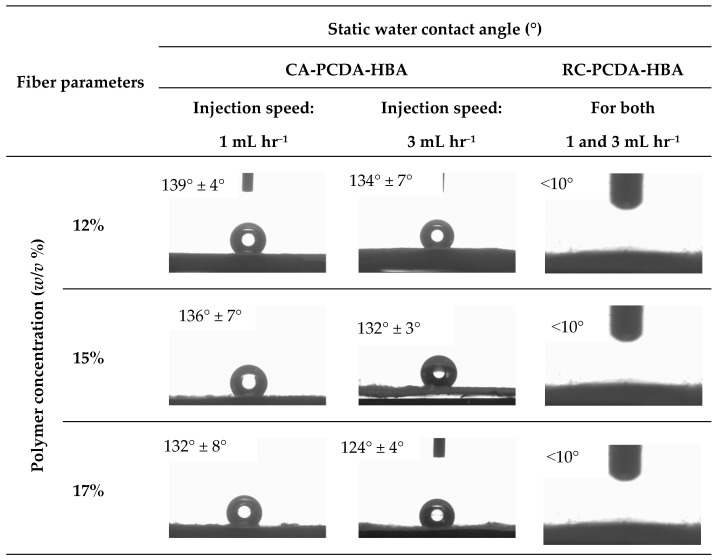
Static water contact angle of the CA-PCDA-HBA and RC-PCDA-HBA nanocomposite fibers.

We further studied the effectiveness of the hydrolysis treatment in converting the hydrophobic CA-PCDA-HBA composite fibers into hydrophilic RC-PCDA-HBA composite fibers via contact angle measurements. Figure 5 presents the contact angles of the fibers under study before and after hydrolysis treatment. The CA-PCDA-HBA composite fibers exhibited contact angles of 139°, 136°, and 132°, respectively, for 12, 15, and 17% concentrations at 1 mL h^−1^ injection speed, pointing out a gradual decrease in contact angle with increasing polymer concentration. On the other hand, at 3 mL h^−1^ injection speed, the fibers exhibited contact angles of 134°, 132°, and 124°, respectively, for 12, 15, and 17% concentrations, indicating a similar trend to the 1 mL h^−1^ injection speed. An increased amount of filler, PCDA-HBA, in the matrix polymer at higher concentrations helped reduce the contact angle. The variations in contact angle might also be related to the morphology (variation in porosity) of the fibers. The RC-PCDA-HBA fiber surfaces are smooth and homogeneous, as can be seen from a closer look at the high magnification images in Figure 2.

The PDA macro molecules are classified as electrical insulators with room temperature conductivities in the range 10^−16^–10^−12^ Ω^−1^ cm^−1^ [38]. Therefore, PCDA-HBA macromolecules did not generate much mobility during the electrospinning process and remained closer to the center of the fiber composite due to nearly zero conductivity. On the other hand, the charged cellulose acetate ions were pushed to the edge of the solution jet due to the high electrostatic repulsive force, making the fibers look homogeneous from the surface. With an increased amount of PCDA-HBA in the fiber matrix, the amount of charged acetate group on the fiber surface is reduced, leading to a gradual decrease in the contact angle. Due to the removal of acetate groups and the development of abundant hydroxyl groups via hydrolysis, the fiber composites, irrespective of concentration and injection speed, became hydrophilic and exhibited contact angles less than 10°. Therefore, the RC-PCDA-HBA is suitable for sensing applications, especially for OP sensing.

#### 3.3.3. Tensile Properties of the Nanocomposite Fibers

**Figure 6:**
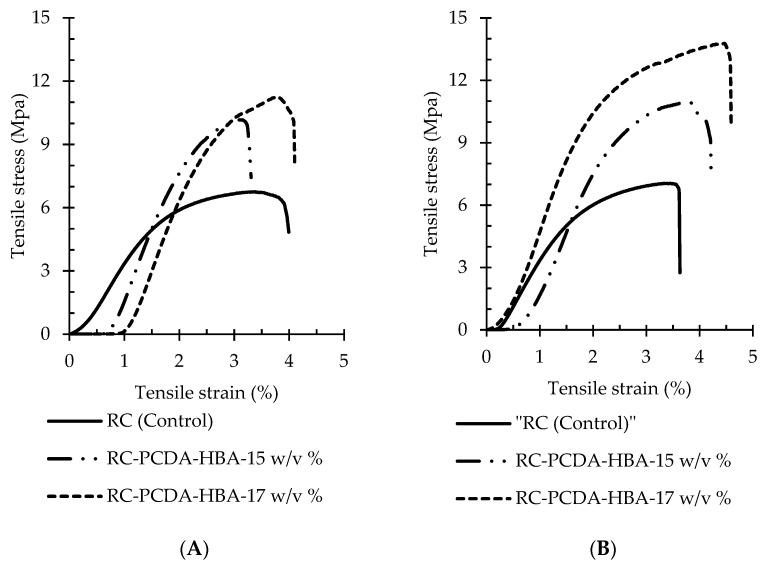
Typical stress–strain curves of the pristine RC (control) and RC-PCDA-HBA electrospun NF composites developed at an injection speed of 1 mL·h^−1^ (**A**) and 3 mL·h^−1^ (**B**).

The sensing materials are expected to be employed in conjunction with regular work clothes. Therefore, appropriate mechanical properties, including tensile strength, elongation, and Young’s modulus, of the NF composite materials are highly essential. The typical stress–strain curves of the pristine RC (control) and RC-PCDA-HBA electrospun NF composites are presented in Figure 6, and the mechanical properties are summarized in Table 2.

The addition of PCDA-HBA altered the mechanical properties of the electrospun fiber composites. In general, improved mechanical properties were observed in the nanocomposite fibers with the gradual addition of PCDA-HBA into the RC matrix. A more than 40% increase in tensile strength was observed in the fibers due to the addition of PCDA-HBA (15 *w/v* %) into the fiber matrix (RC). Moreover, the composites became even stronger after increasing the amount of PCDA-HBA as in the 17 *w/v* % concentration. A similar trend was observed for Young’s modulus. Any large change in breaking elongation in the composite fibers in comparison to the pristine RC fibers was not seen. However, an increasing tendency in elongation was evident with the increase of filler PCDA-HBA. Moreover, the thicker fibers yielded at 3 mL h^−1^ (Figure 6B) exhibited higher strength, elongation, and higher modulus than the fibers yielded at 1 mL h^−1^ (Figure 6A).

Enhancement of the mechanical properties can be attributed to the additional hydrogen bond strength of the filler PCDA-HBA with the matrix polymer. Moazeni and fellow researchers reported a decrease in textile strength and increased elongation and modulus due to the addition of PCDA into hydrophobic polyvinylidene fluoride (PVDF) matrix [28]. However, with the addition of PCDA into the hydrophilic polyethylene oxide (PEO) matrix, an increase in tensile strength was reported by the Li group [38]. The rigid macromolecular chains of PCDA-HBA may be the reason for the increased strength and modulus of the fiber composites. The elongation has not been largely improved due to the reduced flexibility of PCDA-HBA. However, the composites exhibited comparable mechanical properties of the textile structures [35].

#### 3.3.4. Application of PCDA-HBA for Sensing of OP Compounds

**Figure 7:**
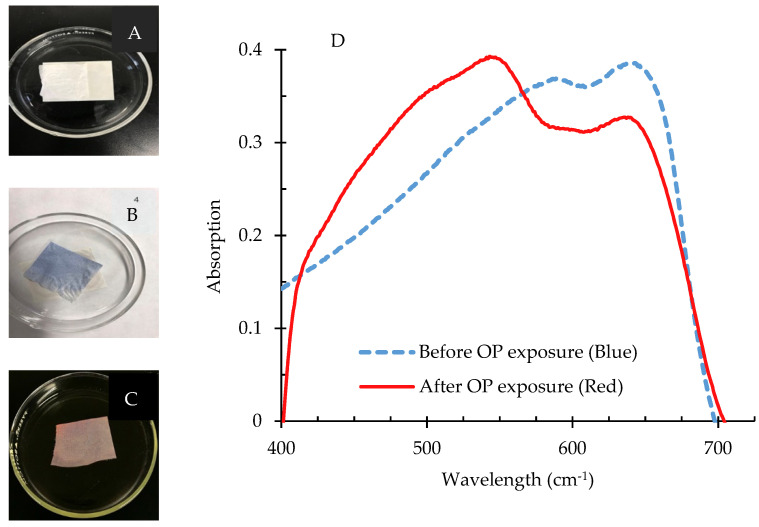
Application of electrospun nanofiber composites for the detection of OP compound, DFP. (**A**) represents white NF composite before photopolymerization, (**B**) exhibits blue colored composite after 254nm UV polymerization, and (**C**) exhibits composites turned red after exposure to DFP. The UV–Vis spectra of the composites before and after color change is exhibited in (**D**).

The detection of the OP compound diisopropyl fluorophosphate (DFP) was tested with RC NF composites containing PDA-HBA and is demonstrated in Figure 7. The white RC-PCDA-HBA NF composites (Figure 7A) did not exhibit any peak in the visible range due to the absence of color; therefore, a spectrum was not included in Figure 7D. Upon photopolymerization, the RC-PDA-HBA composites started turning blue (Figure 7B) within seconds and exhibited a maximum absorption peak around 650 nm, indicating the formation of the π-conjugated backbone of the PDA structure [2,5]. The blue color was found stable for weeks under ambient conditions.

Upon addition of DFP/THF (*Tetrahydrofuran*) solution at a pH of 9.0, the composites started changing color from blue to red/pink within 1 minute (Figure 7C), which was visible to the naked eye. Due to the interaction of the composites with DFP, the absorption peak at 550 nm increased to the maximum with a concurrent decrease in the absorption peak at 650 nm, resulting in the blue-to-red/pink color shift. The color change from blue to red/pink is believed to be due to the chemical reaction of the hydroxyl groups of the PDA ester with DFP, where a primary hydroxyl group is converted into a phosphate ester, causing an increased strain on the conjugated backbone of PDAs [5,39]. Zhang et al. employed density functional theory (DFT) to understand the mechanism of the color change and found that the interactions between ester and OP induced twisting of the π-conjugated PDA backbone to generate the colorimetric change [2]. The irreversible color change from blue to red/pink due to OP sensing was evident to the naked eye and is suitable for analysis by a UV–Vis spectrophotometer. A detailed study on the sensitivity and selectivity of the RC NF composites is under development and will be reported shortly.

## 4. Conclusions

Aldehyde-functionalized PCDA ester, PCDA-HBA, was synthesized via a novel green route for colorimetric sensing of organophosphate (OP) compounds. PCDA-HBA was successfully incorporated into the cellulose acetate (CA) matrix via electrospinning. Polymer concentration and injection speed were found critical for the electrospinning of defect-free nanofibers. A low injection speed along with a low concentration of polymer solution promoted beaded fibers. A high polymer concentration and high injection speed promoted larger fiber diameters. The NF composites were then chemically hydrolyzed to convert CA composites into regenerated cellulose (RC) composites. The FT-IR results exhibited the generation of a large number of hydroxyl groups on the NF composites, which makes the fiber suitable for OP sensing. Moreover, the added functional group into the PCDA macromolecule was not affected by the hydrolysis process. The contact angle measurements confirmed the regeneration of the RC. Improved mechanical properties were observed in the NF composites with the gradual addition of the filler PCDA-HBA into the polymer matrix. The tensile strength was found to be increased by about 40% due to the addition of PCDA-HBA. The Young’s modulus increased with the increase of fiber diameter. The breaking elongation was not affected due to the addition of filler PCDA-HBA. The NF composites could detect the presence of the OP compound DFP within a minute upon exposure and exhibited the detection via a color transition from blue to red. This research study opens a new window toward developing a solid-state sensing platform for OP compounds and contributes to protecting human health and saving lives.

## Figures and Tables

**Table 1:** Optimization of electrospinning processing parameters.

Solution Concentration	Voltage	Distance	Injection Speed
(% *w*/*v*)	(kV)	(cm)	(mL h^−1^)
12	15	10	1
12	15	10	3
15	15	10	1
15	15	10	3
17	15	10	1
17	15	10	3

**Table 2:** Tensile properties of the nanocomposite fibers.

Fiber Parameters			Tensile Properties of the Nanocomposie Fibers		
			Tensile Strength (MPa)	Young’s Modulus (MPa)	Breaking Elongation, %
**RC**	15 *w/v* %	1 mL h^−1^	6.68 ± 1.2	384 ± 86	3.0 ± 0.8
		3 mL h^−1^	7.72 ± 1.5	405 ± 94	4.0 ± 0.9
**RC-PCDA-HBA**	15 *w/v* %	1 mL h^−1^	10.08 ± 1.4	710 ± 45	3.7 ± 0.8
		3 mL h^−1^	11.44 ± 1.7	712 ± 183	4.6 ± 0.9
	17 *w/v* %	1 mL h^−1^	11.71 ± 2.5	718 ± 82	4.1 ± 1.25
		3 mL h^−1^	14.26 ± 0.9	749 ± 114	5.26 ± 0.9

## Data Availability

The data that support the findings of this study are available from the corresponding author upon reasonable request.
